# Effect of preoperative PI-RADS assessment on pathological outcomes in patients who underwent radical prostatectomy

**DOI:** 10.1186/s40644-023-00619-x

**Published:** 2023-11-26

**Authors:** Qianyu Peng, Lili Xu, Gumuyang Zhang, Daming Zhang, Jiahui Zhang, Xiaoxiao Zhang, Xin Bai, Li Chen, Zhengyu Jin, Hao Sun

**Affiliations:** 1 Department of Radiology, State Key Laboratory of Complex Severe and Rare Disease, Peking Union Medical College Hospital, Peking Union Medical College, Chinese Academy of Medical Sciences, Shuaifuyuan No.1, Wangfujing Street, Dongcheng District, Beijing, 100730 China; 2 National Center for Quality Control of Radiology, Shuaifuyuan No.1, Wangfujing Street, Dongcheng District, Beijing, 100730 China

**Keywords:** Magnetic resonance imaging, Prostate imaging–reporting and data system, Radical prostatectomy, Positive surgical margins, Pathological outcomes

## Abstract

**Objective:**

To assess the effect of preoperative MRI with standardized Prostate Imaging–Reporting and Data System (PI-RADS) assessment on pathological outcomes in prostate cancer (PCa) patients who underwent radical prostatectomy (RP).

**Patients and methods:**

This retrospective cohort study included patients who had undergone prostate MRI and subsequent RP for PCa between January 2017 and December 2022. The patients were divided into the PI-RADS group and the non-PI-RADS group according to evaluation scheme of presurgery MRI. The preoperative characteristics and postoperative outcomes were retrieved and analyzed. The pathological outcomes included pathological T stage (pT2 vs. pT3–4) and positive surgical margins (PSMs). Patients were further stratified according to statistically significant preoperative variables to assess the difference in pathological outcomes. A propensity score matching based on the above preoperative characteristics was additionally performed.

**Results:**

A total of 380 patients were included in this study, with 201 patients in the PI-RADS group and 179 in the non-PI-RADS group. The two groups had similar preoperative characteristics, except for clinical T stage (cT). As for pathological outcomes, the PI-RADS group showed a significantly lower percentage of pT3–4 (21.4% vs. 48.0%, p < 0.001), a lower percentage of PSMs (31.3% vs. 40.9%, p = 0.055), and a higher concordance between the cT and pT (79.1% vs. 64.8%, p = 0.003). The PI-RADS group also showed a lower proportion of pT3–4 (p < 0.001) in the cT1–2 subgroup and the cohort after propensity score matching. The PSM rate of cT3 patients was reduced by 39.2% in the PI-RADS group but without statistical significance (p = 0.089).

**Conclusions:**

Preoperative MRI with standardized PI-RADS assessment could benefit the decision-making of patients by reducing the rate of pathologically confirmed non-organ-confined PCa after RP and slightly reducing the PSM rate compared with non-PI-RADS assessment.

**Supplementary Information:**

The online version contains supplementary material available at 10.1186/s40644-023-00619-x.

## Introduction

Prostate cancer (PCa) is the most common cancer among men in over one-half of the countries in the world [1]. In patients with organ-confined PCa, radical prostatectomy (RP) remains the first-line treatment option [2]. Its positive therapeutic effects have been confirmed in recent years [3, 4]. However, there is a risk of upstaging after RP, that is, a preoperatively diagnosed localized PCa (cT1–2) could get a higher stage (pT3–4) at histological examination after RP [5, 6]. These patients have a higher risk of biochemical failure than pT1–2 patients [7]. Positive surgical margins (PSMs) are another important adverse pathological outcome associated with an increased risk of biochemical recurrence and tumor metastasis in patients who have undergone RP [8]. PSMs usually necessitate further adjuvant treatments (androgen-deprivation therapy and/or radiation therapy) [9]. In relatively high-risk cases, neoadjuvant treatment has shown the possibility of achieving local control, which presents the opportunity for surgery [10]. Therefore, identifying patients at high risk for locally advanced PCa and PSMs prior to surgery is critical for treatment decision-making.

At present, magnetic resonance imaging (MRI) is the imaging modality of choice to assess the localization and stage of PCa [11]. A recently published meta-analysis has shown that preoperative MRI results in a change in the decision regarding surgical template in 35% of all patients [12]. Retrospective studies have indicated that preoperative prostate MRI allows for doctors to develop a more optimal surgical plan and reduces the rate of PSMs [12–14]. Nevertheless, these studies varied considerably in interpretation of MRI images. Traditional radiology reports contain unstructured free text in narrative language, which hinders the information transfer and reduces the clarity of the report [15]. In an attempt to improve the interpretation of prostate MRI and the image acquisition techniques standards for global harmonization, the European Society of Urological Surgery (ESUR) established Prostate Imaging–Reporting and Data System version 1 (PI-RADS v1) in 2012 [16]. The PI-RADS was updated to version 2.1 (PI-RADS v2.1) in 2019 [17]. To date, numerous studies have verified the application value of PI-RADS in cancer detection [15, 18, 19]. However, data on potential surgical benefits of the use of standardized PI-RADS reports in preoperative MRI are lacking.

Thus, the purpose of this study was to analyze the effect of preoperative MRI with standardized PI-RADS assessment on the pathological outcomes in patients who underwent RP.

## Materials and methods

### Patients

**Fig. 1:**
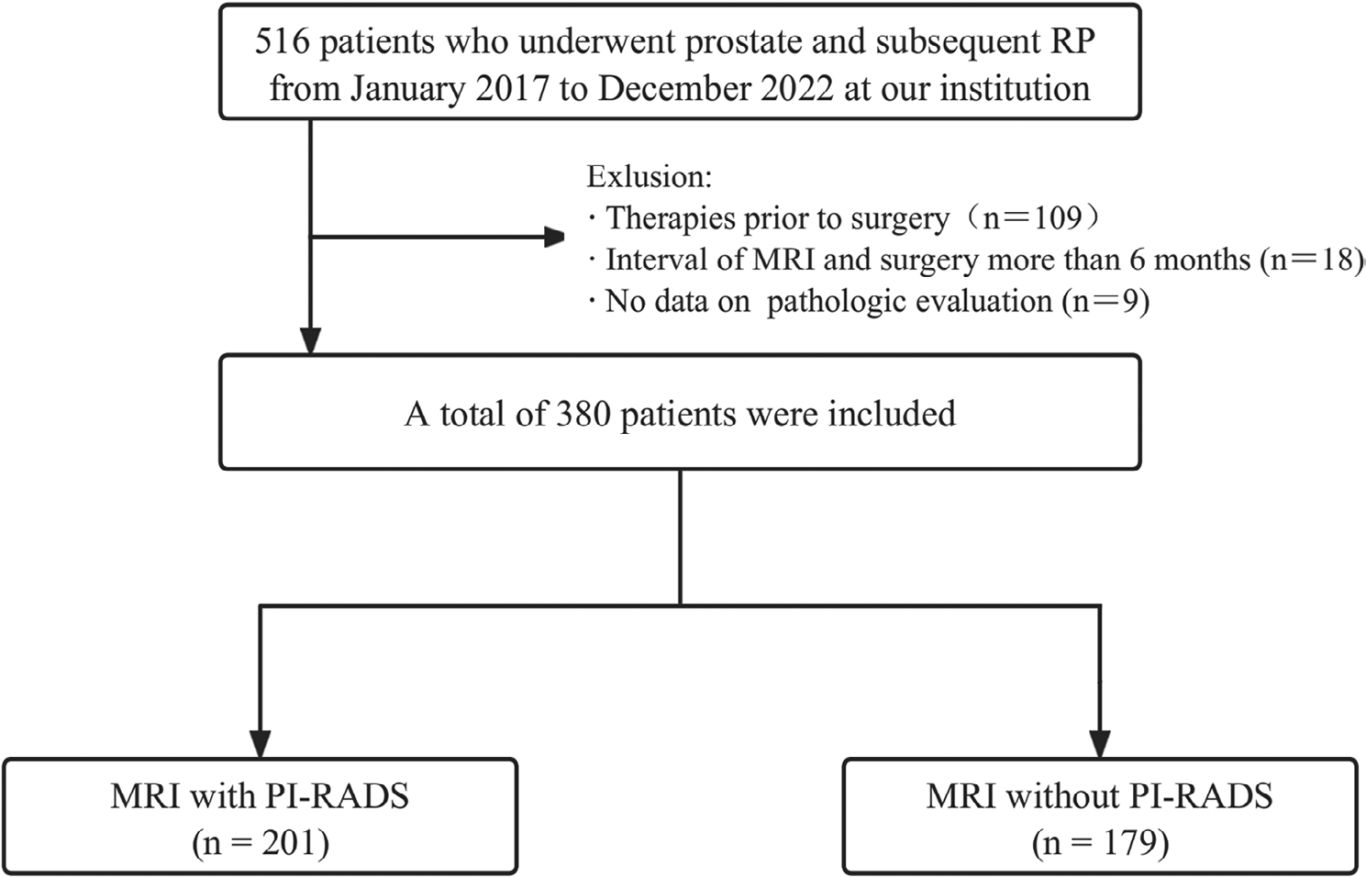
Flowchart of the patient recruitment in this study

This retrospective study was approved by the institutional review board of our institution. Consecutive pathologically confirmed PCa patients who had undergone preoperative prostate MRI followed by RP at our institution between January 2017 and December 2022 were retrospectively enrolled. The exclusion criteria were as follows: (1) interval between MRI and surgery < 6 months; (2) therapies (such as neoadjuvant treatment and a history of prostate-related surgery) received prior to surgery; and (3) incomplete pathological data for evaluation. Figure 1 shows the flowchart for the recruitment process of patients in this study.

### Preoperative clinical, radiological, and histopathological data collection

Clinical, radiological, and preoperative histopathological parameters were collected from the medical and pathology records. The clinical data included age, prostate-specific antigen (PSA) level, cT stage, and surgeon’s experience. From MRI reports, we collected the information as to whether the PI-RADS assessment was performed or not, and the PI-RADS category for the index lesion (for MRI with PI-RADS group). In addition, we recorded whether the suspicious lesion of PCa was indicated in MRI reports. The biopsy regions and biopsy ISUP grade were extracted from the preoperative pathological reports.

### Imaging protocol and analysis

For patients with PI-RADS assessment in our institution, the examinations were performed using 3.0-T MRI (GE750, GE Healthcare, Milwaukee, WI). The imaging protocol of MRI at our hospital followed the PI-RADS recommendation and included T1-weighted imaging (T1WI), T2-weighted imaging (T2WI), diffusion-weighted imaging (DWI), dynamic contrast-enhanced (DCE) imaging, and apparent diffusion coefficient (ADC). The detailed acquisition parameters are shown in Supplementary Table S1. As for the remaining patients with PI-RADS assessment, their MRI examinations were performed at other institutions; therefore, the details of the imaging parameters were unknown. The majority of patients in the MRI without PI-RADS group underwent MRI scanning in external institutions. For patients in the MRI without PI-RADS group who underwent MRI scanning at our institution, the minimum standard imaging protocol was biparametric, including T2WI, DWI, and ADC. Three radiologists with different experiences in interpreting prostate MRI (H.S., with 9 years of experience, > 4 500 cases; G.Z., with 6 years of experience, > 1 500 cases; and D.Z. with 4 years of experience, > 1 000 cases) evaluated the MRI images at our institution and determine the PI-RADS category for each lesion.

During the course of this study, the standardized reports in the MRI with PI-RADS group were considered to refer to PI-RADS v2 or PIRADS v2.1.

### Pathological evaluation

The final postoperative histopathological diagnosis was defined as the standard reference. All of the RP specimens were processed in accordance with clinical routine with the thickness of 0.4 cm and assessed by senior pathologists. The pathologists further recorded the number and location of tumors, presence of PSMs, and final pathological stage (pT) for each patient. PSMs were defined as the presence of PCa cells at the inked margins [20]. Pathological grading of biopsy and postoperative specimens was evaluated in accordance with the 2005 ISUP consensus on Gleason grading [21]. The postoperative upstaging cases were defined as the preoperatively diagnosed localized PCa (cT1–2) that obtained a higher stage (pT3–4) at histological examinations after RP.

### Statistical analysis

Differences in the preoperative clinical, radiological, and histopathological parameters and postoperative pathological parameters between the PI-RADS group and the non-PI-RADS group were analyzed using the chi-square test, Fisher’s exact test, and the Mann–Whitney *U* test at the significance level of 0.05. The patients were further stratified by the statistically significant preoperative variables to assess the difference in the pathological outcomes between the two groups. To attempt to reduce selection bias or confounding factors from differences in baseline variables, one-to-one nearest neighbor matching using the propensity score matching technique was conducted with age, PSA level (< 10 or ≥ 10 ng/dL), ISUP grade, cT, and surgery level as the matching variables. Statistical analysis was performed using R software (https://www.R-project.org/) and SPSS 25.0 (IBM, Armonk, NY).

## Results

### Preoperative clinicopathological features

A total of 380 patients were finally enrolled in this study, including 201 (52.9%) patients who had a PI-RADS assessment and 179 (47.1%) patients who did not. In the PI-RADS group, 76.6% (n = 154) of the assessments were performed at our hospital and 23.4% (n = 47) were performed at other institutions. There were only 16 patients (8.9%) without PI-RADS assessment at our hospital. The clinicopathological characteristics of the patient cohort are presented in Table 1. The preoperative cT stage was significantly different between the two groups, with 84.6% of cT1–2 patients in the PI-RADS group and 96.1% of cT1–2 in the non-PI-RADS group (p < 0.001). Age, PSA, ISUP group in biopsy, and surgical experience did not significantly differ between the two groups (all p > 0.05).

**Table 1:** Clinicopathological features of the patients

Variables		Overall cohort (n = 380)	PI-RADS (n = 201)	Non-PI-RADS (n = 179)	*p*
Preoperative	Age (year)^*^	66 (62–70)	67 (63–70)	65 (61–70)	0.085
	PSA (ng/mL)				0.062
	≤ 10	205 (53.9)	118 (58.7)	87 (48.6)	
	> 10	175 (46.1)	83 (41.3)	92 (51.4)	
	Clinical T stage				< 0.001
	cT1-2	342 (90.0)	170 (84.6)	172 (96.1)	
	cT3	38 (10.0)	31 (15.4)	7 (3.9)	
	ISUP grade				
	1	116 (30.5)	62 (31.0)	54 (30.2)	0.187
	2	94 (24.7)	54 (27.0)	40 (22.3)	
	3	76 (20.0)	44 (22.0)	32 (17.9)	
	≥ 4	94 (24.7)	41 (20.0)	53 (29.6)	
	Surgical experience				0.954
	< 100	268 (70.5)	141 (70.1)	127 (70.9)	
	≥ 100	112 (29.5)	60 (29.9)	52 (29.1)	
	PI-RADS				
	2		6 (3.0)		
	3		28 (13.9)		
	4		102 (50.7)		
	5		65 (32.3)		
	Indication of suspicious lesions	345 (93.1)	195 (97.0)	150 (83.8)	< 0.001
	Correct preoperative staging	275 (72.3)	159 (79.1)	116 (64.8)	0.003
Postoperative	Pathological T stage				< 0.001
	pT2	269 (70.8)	158 (78.6)	111 (62.0)	
	pT3-4	111 (29.2)	43 (21.4)	68 (48.0)	
	Upstaging	89 (23.4)	27 (13.4)	62 (34.6)	< 0.001
	PSM	136 (35.8)	63 (31.3)	73 (40.9)	0.055

Note—Unless otherwise indicated, data are number (percentages). PSA = prostate-specific antigen; ISUP = international society of urological pathology; PSM = positive surgical margin; PI-RADS = Prostate Imaging-Reporting and Data System

^*^ Data are median (interquartile range [IQR])

The detection rate of suspicious lesions was higher in the PI-RADS group (97.0% vs. 83.8%, p < 0.001), and the accuracy of preoperative clinical staging in the PI-RADS group was higher than the non-PI-RADS group (79.1% vs. 64.8%, p = 0.003).

## Postoperative pathological outcomes

The analysis of pathological outcomes showed a lower percentage of patients with pT3–4 status in the PI-RADS group (21.4% vs. 48.0%, p < 0.001). The percentage of upstaging after RP was also lower in the PI-RADS group (13.4% vs. 34.6%, p < 0.001). The PSM rates were lower in the PI-RADS group than the non-PI-RADS group (31.3% vs. 40.9%), but the difference was not statistically significant (p = 0.055).

Because the cT stage was the only statistically significant preoperative variable between the two groups, further subgroup analysis was performed based on this factor as shown in Table 2. Among the patients with cT1–2, the proportion of patients with pT3–4 status was lower in the PI-RADS group (15.9% vs. 36.1%, p < 0.001). In the cT3 subgroup, the rate of PSMs was lower in the PI-RADS group than in non-PI-RADS group (32.2% vs. 71.4%, p = 0.089). Also in this subgroup, the pathological stage was not significantly different between the two groups (p = 0.241).

**Table 2:** Subgroup analysis based on clinical T stage

Subgroup	Postoperative features	PI-RADS (n = 201)	Non-PI-RADS (n = 179)	*p*
cT1-2	Pathological T stage			< 0.001
	pT2	143 (84.1)	110 (63.9)	
	pT3-4	27 (15.9)	62 (36.1)	
	PSM	53 (31.2)	68 (39.5)	0.133
cT3	Pathological T stage			0.241
	pT2	15 (48.4)	1 (14.3)	
	pT3-4	16 (51.6)	6 (85.7)	
	PSM	10 (32.2)	5 (71.4)	0.089

Note—Unless otherwise indicated, data are number (percentages). PSM = positive surgical margin; PI-RADS = Prostate Imaging-Reporting and Data System

### Postoperative pathological outcomes after propensity score matching

The clinicopathological features of the one-to-one nearest neighbor–matched cohort are shown in Table 3. One-to-one nearest neighbor matching generated 161 matched pairs with no significant difference in age, PSA level, ISUP grade, cT, and surgeon’s experience (all p > 0.05). The detection rate of suspicious lesions was higher in the PI-RADS group than the non-PI-RADS group (96.9% vs. 82.0%, p < 0.001).

**Table 3:** Clinicopathological features of the one-to-one nearest neighbor–matched cohort

Variables		PI-RADS (n = 161)	Non-PI-RADS (n = 161)	*p*
Preoperative	Age (year)*	66.02 (5.81)	65.82 (6.09)	0.765
	PSA (ng/mL)			0.215
	≤10	98 (60.9)	86 (53.4)	
	>10	63 (39.1)	75 (46.6)	
	Clinical T stage			
	cT1-2	154 (95.7)	154 (95.7)	1.000
	cT3	7 (4.3)	7 (4.3)	
	ISUP grade			0.457
	1	52 (32.3)	54 (33.5)	
	2	47 (29.2)	38 (23.6)	
	3	33 (20.5)	30 (18.6)	
	≥ 4	29 (18.0)	39 (24.2)	
	Surgical experience			0.718
	<100	109 (67.7)	113 (70.2)	
	≥100	52 (32.3)	48 (29.8)	
	Indication of suspicious lesions on MRI	156 (96.9)	132 (82.0)	< 0.001
Postoperative	Pathological T stage			0.001
	pT2	132 (82.0)	105 (65.2)	
	pT3-4	29 (18.0)	56 (34.8)	
	Upstaging	25 (15.5)	50 (31.1)	0.002
	PSM	50 (31.1)	62 (38.5)	0.198

Note—Unless otherwise indicated, data are number (percentages). PSA = prostate-specific antigen; ISUP = international society of urological pathology; PSM = positive surgical margin; PI-RADS = Prostate Imaging-Reporting and Data System

^*^ Data are mean (standard deviation [SD])

In the nearest neighbor–matched patient cohort, a lower percentage of patients with pT3–4 status (18.0% vs. 34.8%, p = 0.001) and with upstaging (15.5% vs. 31.1%, p = 0.002) was found in the PI-RADS group. The PSM rate was 31.1% in the PI-RADS group and 38.5% in the non-PI-RADS group (p = 0.198).

## Discussion

The present study reported that preoperative prostate MRI with PI-RADS assessment might have a potential impact on pathological outcomes. In this large series of patients who underwent RP, preoperative PI-RADS assessment reduced the proportion of patients with pT3–4 status, decreased the cases of upstaging after RP, and improved the accuracy of preoperative clinical staging. The PSM rate in the PI-RADs group was lower than that in the non-PI-RADS group, although the difference was not statistically significant.

The PI-RADS assessment increased the cancer detection rate compared with the non-PI-RADS assessment pathway. A retrospective audit [22] demonstrated a statistically significant increase in prostate tumor detection by multiparametric magnetic resonance imaging (mpMRI) combined with PI-RADS v2 reporting (detection rates of the dominant tumor nodule: 67% for mpMRI vs. 91% for mpMRI combined with PI-RADS v2). The results of our study showed that preoperative MRI with standardized PI-RADS assessment improved the detection rate of suspicious lesions, which increased from 83.8 to 97.0% in the entire cohort and from 82.0 to 96.9% in the nearest neighbor–matched cohort. This improvement in tumor detection can be at least partly attributed to the structured scoring system of PI-RADS with clear guidelines for using dominant sequences in the peripheral zone and transitional zone. And, the published literature has demonstrated high inter-reader agreement between experienced readers and those with at least 1 year of experience and high sensitivity in high-grade index lesion PCa on mpMRI [19, 23]. These results encourage the application of PI-RADS assessment in clinical practice.

The correct distinction between organ-confined disease and locally advanced stage has a large impact not only on the prognosis, but also on treatment planning. According to Haug et al. [14], the number of locally advanced high-risk patients who underwent surgery and had received pre-biopsy MRI of the prostate (MRI-P) has declined after 2016. The result suggests that cT3–4 stages in patients with MRI-P have shifted the treatment toward radiotherapy. During the same period, the PSM rate of positive pT3 tumors decreased steadily. In our study, the PI-RADS group showed a lower proportion of patients with pT3–4, which was verified in the subgroup analysis of the patients stratified by cT and the nearest neighbor–matched cohort. This suggests that MRI based on PI-RADS assessment provided knowledge of tumor location and extent preoperatively to reduce the number of patients who are not eligible for the priority RP. In a recent retrospective study by Jäderling et al. [13], compared with postoperative histopathology, there was 80% accuracy of preoperative staging (exact match 61% and approximate match 19%) in all of the patients assessed by PI-RADS version 1 (360 patients) and PI-RADS version 2 (197 patients). Our study reported a similar concordance between the cT and pT to the study by Jäderling et al. (79.1% vs. 80%). Additionally, our results showed a lower accuracy of preoperative clinical staging in the non-PI-RADS group than in the PI-RADS group (79.1% vs. 64.8%). In the non-PI-RADS group, most of the patients with incorrect clinical stage demonstrated a pathologically confirmed upstaging after RP (upstaging: 34.6% [62/179]; downstaging: 0.6% [1/179]). The reasons might be that without standardized reporting, radiologists are likely to miss some important information about cancer staging and, therefore, tend to underestimate the aggressiveness of cancer. While in the PI-RADS group, the percentage of incorrectly staged patients was significantly lower than in the non-PI-RADS group (20.9% [42/201] vs. 35.2% [63/179]), especially the pathologically confirmed upstaging patients (13.4% [27/201] vs. 34.6% [62/179]). Interestingly, the percentage of downstaging ones after RP is relatively higher than the non-PI-RADS group (7.5% [15/201] vs. 0.6% [1/179]). PI-RADS guideline proposed some risk features for evaluating extraprostatic extension (EPE) of PCa (T3), however, a standardized assessment scheme regarding EPE is lacking. Although many studies have tried to propose classification systems for EPE evaluation, the reported positive prediction value raged from 0.74 to 0.875, which means by taking those criteria, some patients might be downstaging by the pathology [24]. Even with relatively high clinical staging, these patients were considered eligible to undergo surgery after thorough clinical evaluation, and the PSM rate in this group was reduced to a certain extent compared with the non-PI-RADS group (32.2% [10/31] vs. 71.4% [5/7]), which may have a potential impact on pathological outcomes [22]. Therefore, on one hand, modifying the staging criteria on MRI is necessary for future studies, on the other hand, the urologists should take the standardized reports into consideration carefully by combing with other clinical factors. The results of this study showed a higher proportion of patients with upstaging after RP (13.4% vs. 34.6%, p < 0.001) in the non-PI-RADS group, which was also verified in the matched cohort (15.5% vs. 31.1%, p = 0.002). Therefore, for better PCa management and treatment decision-making, a standardized prostate MRI report—PI-RADS assessment—should be recommended.

Preoperative MRI with PI-RADS assessment seems to reduce the PSM rate in PCa patients who underwent RP to some extent. In our study, the overall PSM rate was 35.8%, which is equivalent to previous reports of 16–46% [25, 26]. Many studies have examined whether there is a reduction of the PSM rate in patients who underwent preoperative MRI or not. Haug et al. found that mpMRI reduced the PSM rates compared with preoperative non-mpMRI [14]. Jäderling et al.‘s [13] study also suggested that preoperative prostate mpMRI affected the degree of nerve-sparing surgery and reduced the PSM rate compared with preoperative non-MRI. According to a meta-analysis by Patel et al. [27], a similar magnitude in the reduction of the PSM rate of about 5% for patients receiving mpMRI was observed, which reached statistical significance with very low heterogeneity across studies. Despite the widely investigated impact of preoperative MRI for PSMs, the studies focusing on PI-RADS assessment for PSMs are rare. In our study, all of the patients underwent preoperative MRI examination, and the discussion centered on the influence of PI-RADS evaluation on the surgical results. Our study showed an overall 9.6% reduction in PSMs in the patients with PI-RADS assessment, 7.4% in the matched cohort, 8.3% in the cT1–2 subgroup, and 39.2% in the cT3 subgroup, although the reduction did not reach statistical significance. The reduction of the PSM rate in the PI-RADS group could be attributed to the lower percentage of locally advanced PCa in the same group relative to the non-PI-RADS group, given that many studies have demonstrated that extraprostatic extension of PCa is an important risk factor for PSMs. Although the reduction in the PSM rates was not statistically significant, we believe that there could still be potential benefits. This suggests that the value of PI-RADS is not only to provide surgeons with accurate information about tumor location, but also to perform accurate preoperative staging to benefit surgical outcomes.

There are several limitations to this study. First, the single-center, retrospective cohort study may have introduced some selection bias. Second, a part of the MRI collection in this study was conducted in an external hospital, which is why the details of the scan parameters were not available, and there may be some variation in the interpretation of PI-RADS. Furthermore, our retrospective analysis lacked the complete insight into the surgical protocol decisions of individual patients. As far as we know, the surgical approach is also a factor affecting PSMs. Further studies about the relative contribution of the standardized PI-RADS to the PSM rate are needed.

## Conclusion

Prostate MRI with standardized PI-RADS assessment improves the detection rate of suspicious lesions and the accuracy of preoperative clinical staging. With PI-RADS assessment, the proportion of patients with non-organ-confined PCa undergoing RP decreases, and the PSM rate is slightly reduced compared with non-PI-RADS assessment.

### Electronic supplementary material

Below is the link to the electronic supplementary material.

Supplementary Material 1

## Data Availability

The datasets used and analyzed during the current study are available from the corresponding author on reasonable request.

## Abbreviations

PSMs: Positive surgical margins
ISUP: International Society of Urological Pathology
mpMRI: Multiparametric magnetic resonance imaging
PI-RADS: Prostate Imaging-Reporting and Data System
PCa: Prostate cancer
PSA: Prostate-specific antigen
RP: Radical prostatectomy
