# Supplementary material for: Effect of preoperative PI-RADS assessment on pathological outcomes in patients who underwent radical prostatectomy
Source: Cancer Imaging. 2023 Nov 26;23:113. doi: 10.1186/s40644-023-00619-x (PMC10680237; doi:10.1186/s40644-023-00619-x)
Supplement: Supplementary file 1 — Supplementary Material 1 [file 40644_2023_619_MOESM1_ESM.pdf]

Supplementary material

Effect of preoperative PI-RADS assessment on pathological outcomes in patients who underwent radical prostatectomy

Table S1. Sequence parameters for prostate multiparametric MRI.

**Parameters**	**T2WI**	**DWI**	**DCE**
Sequence	FRFSE	SE-EPI	3D-GRE
TR/TE (ms)	4137/86	4200/90	4.3/1.3
Flip angle (degree)	110	90	12
Echo train length	32	1	N/A
Field of view (mm × mm)	270 × 270	360 × 360	400 × 400
Matrix size	288 × 192	128 × 96	320 × 192
Thickness (mm)	3.0	3.0	3.0
Other		b values= 100, 150, 200, 500, 800, 1000, 1500, 2000 mm^2/sec	Temporal resolution <10s, and total scan time of 5 min

TR, repetition time; TE, time echo; FRFSE, fast relaxation fast spin echo; SE-EPI, spin-echo echo planar imaging; 3D-GRE, 3D-gradient echo; DWI, diffusion-weighted imaging; T2WI, T2-weighted imaging; DCE, dynamic contrast-enhanced; ADC, apparent diffusion coefficient.
